# Cell Phone Bans in a National Sample of US Public School Principals

**DOI:** 10.1001/jamahealthforum.2025.4229

**Published:** 2025-10-03

**Authors:** Jonathan Cantor, Ryan K. McBain, Aaron Kofner, Fang Zhang, Alyssa Burnett, Joshua Breslau, Melissa Kay Diliberti, Ateev Mehrotra, Bradley D. Stein, Hao Yu

**Affiliations:** 1 RAND, Santa Monica, California; 2 RAND, Arlington, Virginia; 3 Brigham & Women’s Hospital, Boston, Massachusetts; 4 Harvard Medical School, Boston, Massachusetts; 5 Harvard Pilgrim Health Care Institute, Boston, Massachusetts; 6 RAND, Pittsburgh, Pennsylvania; 7 Brown University School of Public Health, Providence, Rhode Island

## Abstract

This cross-sectional study reports the national prevalence of public-school cell phone bans and how they vary by school characteristics.

## Introduction

The increase in child mental health concerns in the US^1^ has coincided with widespread adoption of digital technologies among youth. Citing concerns about cell phone use and mental health and academic performance^2^ as well as the association between greater screen time and diminished mental well-being,^3^ many school systems and several states have adopted school cell phone bans.^4^ On average, adolescents use their smartphones for 66 minutes during school.^5^ However, no published data exist on the national prevalence or structure of school cell phone policies. This study is the first to our knowledge to report the national prevalence of public-school cell phone bans and how they vary by school characteristics.

## Methods

We surveyed a nationally representative sample of kindergarten through grade 12 public school principals in October 2024 via the RAND American School Leader Panel (ASLP).^6^ Principals reported their 2024-2025 school cell phone policy. The eMethods in Supplement 1 contains question details and categorization. The free-text responses when respondents selected “other” were categorized into one of the other choices. Responses were linked to the National Center for Education Statistics Common Core of Data to examine variation by grade level, school size, racial and ethnic composition, urbanicity, census region, and neighborhood poverty.

We categorized policy strictness from most strict to least strict: (1) a student cannot bring a cell phone to school, (2) a student can bring a cell phone to school but cannot use it when school is in session, (3) a student can bring a cell phone to school and can use it outside of class time, (4) student can bring a cell phone to school and use it in class, with the teacher’s discretion, and (5) this school does not have a cell phone policy. We then conducted an ordinal logistic regression estimating policy strictness and applied ASLP survey weights to ensure national representativeness.

Analyses used Stata version 19.5 (StataCorp). A 2-sided *P* < .05 was used for statistical significance. The study was deemed exempt from review by Harvard Pilgrim Health Care Institute institutional review board as a secondary analysis of deidentified data and followed the STROBE reporting guideline.

## Results

Of the 985 respondents, 96.68% reported their school having a cell phone policy. Elementary schools had higher rates of not allowing a cell phone in school (6.79%) and not allowing a cell phone to be used while school is in session (81.62%) (Figure). In contrast, middle (6.91%) and high schools (23.45%) were more likely to allow student cell phone use when class is not in session (or at the teacher’s discretion) compared with elementary schools (3.80%).

**Figure.**
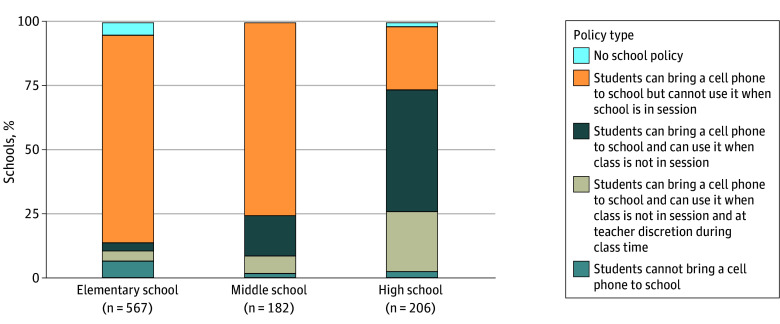
School Cell Phone Policy for 955 Schools That Listed Their School Level With a Cell Phone Policy, 2024-2025 Includes survey respondents based on National Center for Education Statistics grade level. Percentages were calculated using the RAND American School Leader Panel survey weights to be representative of kindergarten through grade 12 public school principals. The survey question was “What is your school’s cell phone policy this year (2024-25)?” School characteristics are from the National Center for Education Statistics Common Core of Data. The question and listed school cell phone policies are included in the eMethods of Supplement 1.

Our regression analysis found middle (odds ratio [OR], 2.27; 95% CI, 1.52-3.40) and high schools (OR, 11.98; 95% CI, 7.83-18.33) (*P* < .001) were more likely to have less strict cell phone policies compared with elementary schools. We also found schools located in low (OR, 1.59; 95% CI, 1.01-2.51; *P* = .04) and medium poverty neighborhoods (OR, 1.64; 95% CI, 1.12-2.40; *P* = .01) were more likely to have less strict policies compared with schools in high-poverty neighborhoods (Table).

**Table.** Association Between School Characteristics and Having a Less Strict Cell Phone Policy (n = 941 Respondents)^a^

School characteristic	Odds ratio (95% CI)	*P* value
Grade level		
Elementary school	1 [Reference]	NA
Middle school	2.27 (1.52-3.40)	<.001
High school	11.98 (7.83-18.33)	<.001
Urbanicity		
Urban	1 [Reference]	NA
Suburban	1.08 (0.76-1.53)	.68
Town or rural	1.18 (0.78-1.78)	.43
Race and ethnicity of student population^b^		
Most students with White race	1 [Reference]	NA
Most students with other race and ethnicity	1.12 (0.78-1.59)	.55
Neighborhood poverty		
High neighborhood poverty (income to poverty ratio ≤200)	1 [Reference]	NA
Middle neighborhood poverty (income to poverty ratio 201-400)	1.64 (1.12-2.40)	.01
Low neighborhood poverty (income to poverty ratio ≥401)	1.59 (1.01-2.51)	.04
School size, No. of students		
≤450	1 [Reference]	NA
>450	1.04 (0.79-1.39)	.76
Census region		
Northeast	1 [Reference]	NA
Midwest	1.09 (0.72-1.65)	.69
South	1.11 (0.71-1.73)	.65
West	1.38 (0.87-2.19)	.18

Abbreviation: NA, not applicable.

^a^ The estimation sample for the regression was restricted to 941 respondents due to missing data for covariates. Ordinal logistic regression was estimated using the RAND American School Leader Panel survey weights to be representative of kindergarten through grade 12 public school principals. The survey question was “What is your school’s cell phone policy this year (2024-25)?” School characteristics are from the National Center for Education Statistics Common Core of Data and the Education Demographic and Geographic Estimates School Neighborhood Poverty Estimates. Outcome measure is a categorical measure with a value of 1 for the response “Students cannot bring a cell phone to school.” A value of 2 is assigned to the response “Students can bring a cell phone to school, but *cannot use it when school is in session.”* A value of 3 is assigned to the response “Students can bring a cell phone to school, and *can use it when class is not in session (eg, during lunch or hallway transition time) but not during class time.”* A value of 4 is assigned to the response “Students can bring a cell phone to school, and *can use it when class is not in session and, at teachers’ discretion, during class time.”* Finally, schools without a phone policy were assigned a value of 5. One school’s response was excluded from the analysis because it was unclassifiable. The question and listed school cell phone policies are included in the eMethods of Supplement 1.

^b^ In the source data file, the specific terms used were “majority White” and “majority students of color” for convenience without further explanation available.

## Discussion

Although nearly all public schools restrict cell phone use, policies vary widely. Bans were less strict in high schools, when students are obtaining greater independence and many may have greater need for cell phones, but also in an age range with higher rates of depression.^1^ Bans were also less strict in low-poverty and medium-poverty neighborhoods compared with high-poverty neighborhoods; the reasons for this pattern warrant further investigation.

This study has limitations. We lack trend data, do not assess nonpublic schools, and cannot determine whether policies are enforced or effective. We also do not investigate consequences of cell phone policies in the academic and mental health outcomes of children. Furthermore, the survey did not address other smart devices, such as smartwatches. Still, to our knowledge, this is the first nationally representative analysis of public school cell phone policies.

## Notes

Supplement 1. **eMethods.** RAND American Educator Panels: Fall 2024 Omnibus Principal Survey

Supplement 2.
Data Sharing Statement
