# Supplementary material for: Cell Phone Bans in a National Sample of US Public School Principals
Source: JAMA Health Forum. 2025 Oct 3;6(10):e254229. doi: 10.1001/jamahealthforum.2025.4229 (PMC12495490; doi:10.1001/jamahealthforum.2025.4229)
Supplement: Supplement 1. — eMethods. RAND American Educator Panels: Fall 2024 Omnibus Principal Survey [file jamahealthforum-e254229-s001.pdf]

Supplemental Online Content

Cantor J, McBain RK, Kofner A, et al. Cell phone bans in a national sample of US public school principals. *JAMA Health Forum*. 2025;6(10):e254229. doi:10.1001/jamahealthforum.2025.4229

eMethods. RAND American Educator Panels: Fall 2024 Omnibus Principal Survey

This supplemental material has been provided by the authors to give readers additional information about their work.

© 2025 Cantor J et al. *JAMA Health Forum*.

eMethods. RAND American Educator Panels: Fall 2024 Omnibus Principal Survey

What is your school's cell phone policy this year (2024-25)?
SELECT ONE RESPONSE

- N/A; our school does not have a cell phone policy
- Students cannot bring a cell phone to school
- Students can bring a cell phone to school, but cannot use it when school is in session
- Students can bring a cell phone to school, and can use it when class is not in session (e.g., during lunch or hallway transition time) but not during class time
- Students can bring a cell phone to school, and can use it when class is not in session and, at teachers' discretion, during class time
- Other (please describe:)

© 2025 Cantor J et al. *JAMA Health Forum*.
