# Supplementary material for: Cell Phone Bans in a National Sample of US Public School Principals
Source: JAMA Health Forum. 2025 Oct 3;6(10):e254229. doi: 10.1001/jamahealthforum.2025.4229 (PMC12495490; doi:10.1001/jamahealthforum.2025.4229)
Supplement: Supplement 2. — Data Sharing Statement [file jamahealthforum-e254229-s002.pdf]

Data Sharing Statement

Cantor. Cell Phone Bans in a National Sample of US Public School Principals. *JAMA Health Forum*. Published October 03, 2025. doi:10.1001/jamahealthforum.2025.4229

Data
Data available: No

Additional Information
Explanation for why data not available: The data can be acquired upon request and after filling out a data use agreement.
